# Supplementary material for: Dynamic of the Soil Microbiota in Short-Term Crop Rotation
Source: Life (Basel). 2023 Feb 1;13(2):400. doi: 10.3390/life13020400 (PMC9961364; doi:10.3390/life13020400)
Supplement: Supplementary file 1 [file life-13-00400-s001.zip › life-2054494-supplementary.pdf]

Supplementary Table S1. Connection between microbial richness (Observed ASVs) and culture of plants, phaseI. ANOVA results.

	Df	SumSq	MeanSq	F	p-value
Culture	5	841473	168295	14.04	2.62e-11
Residuals	154	1845891	11986		

Supplementary Table S2. Connection between Bray-Curtis distances and culture of plants, phase I. PERMANOVA results.

	Df	SumSq	R2	F	p-value
Culture	5	1.2226	0.07602	2.5342	0.001
Residuals	154	14.8587	0.92398		
Total	159	16.0812	1.00000		

Number of permutation – 999.

Supplementary Table S3**.** Connection between microbial richness (Observed ASVs) and culture of plants, phase II. ANOVA results.

	Df	SumSq	MeanSq	F	p-value
SecondatryCulture	4	26067	6517	0.868	0.4852
PrimaryCulture	4	526666	131667	17.538	2.06e-11
SecondaryCulture:PrimaryCulture	16	237302	14831	1.976	0.0197
Residuals	123	923405	7507		

Supplementary Table S4. Connection between Bray-Curtis distances and culture of plants, phase II. PERMANOVA results.

	Df	SumSq	R2	F	p-value
PrimaryCulture	4	0.9838	0.06442	2.1765	0.001
SecondaryCulture	4	0.6474	0.04239	1.7903	0.001
PrimaryCulture:SecondaryCulture	16	1.7086	0.11187	1.1812	0.001
Residual	123	11.9335	0.78133		
Total	147	15.2734	1.00000		

Number of permutation – 999
